# Synthesis and application of Amberlite xad-4 functionalized with alizarin red-s for preconcentration and adsorption of rhodium (III)

**DOI:** 10.1186/1735-2746-9-7

**Published:** 2012-09-18

**Authors:** Hossein Sid Kalal, Homayon Ahmad Panahi, Hassan Hoveidi, Mohammad Taghiof, Mahnaz Taheri Menderjani

**Affiliations:** 1 Nuclear Fuel Cycle Research School, Nuclear Science and Technology Research Institute, Atomic Energy Organization of Iran, Tehran, Iran; 2 Department of Chemistry, Central Tehran Islamic Azad University, Tehran, Iran; 3 Graduate Faculty of Environment, University of Tehran, Tehran, Iran

**Keywords:** Environmental measurement, Solid phase extraction, Amberlite XAD-4, Rhodium, Immobilization

## Abstract

A new chelating resin was prepared by coupling Amberlite XAD-4 with alizarin red-s through an azo spacer, characterized by infra-red spectroscopy and thermal analysis and studied for Rh(III) preconcentration using inductively coupled plasma atomic emission spectroscopy (ICP-AES) for rhodium monitoring in the environment. The optimum pH for sorption of the metal ion was 6.5. The sorption capacity was found 2.1 mg/g of resin for Rh(III). A recovery of 88% was obtained for the metal ion with 1.5 M HCl as eluting agent. Kinetic adsorption data were analyzed by adsorption and desorption times of Rh(III) on modified resin. Scat chard analysis revealed that the homogeneous binding sites were formed in the polymers. The linear regression equation was Q/C = –1.3169Q + 27.222 (R^2^ = 0.9239), for Rh were formed in the SPE sorbent,K_d_ and Q_max_ for the affinity binding sites were calculated to be 0.76 μmol/mL and 20.67 μmol/g, respectively. The equilibrium data and parameters of Rh(III) adsorption on modified resin were analyzed by Langmuir, Freundlich, Temkin and Redlich–Peterson models. The experimental adsorption isotherm was in good concordance with Langmuir and Freundlich models (R^2^ > 0.998) and based on the Langmuir isotherm the maximum amount of adsorption (q_max_) was 4.842 mg/g. The method was applied for rhodium ions determination in environmental samples. with high recovery (>80%).

## Introduction

The interest in ligand immobilized solid phase like silica gel [1,2], organic polymer or copolymers, cellulose [3,4] and polyurethane foam [5] continues because of their several applications, for example in solid phase metal extraction [6], designing hybrid organic–inorganic catalysts [7] and heterogenization of homogeneous catalysts [8]. Solid phase extraction of metal ions present at trace level in environmental samples, high purity materials, biological samples and other complex matrices, makes the analytical techniques possible, such as flame atomic absorption spectrometry (FAAS) and inductive couple plasma atomic emission spectroscopy (ICP-AES). Solid phase extraction is preferable over ion exchange and solvent extraction due to its advantages like selectivity by controlling pH, reusability, high pre concentration factors, durability, versatility and metal loading capacity [9-13].

Adsorption of metal ions is widely used in the removal of contaminants from wastewaters. The design and efficient operation of adsorption processes require equilibrium adsorption data. The equilibrium isotherm plays an important role in predictive modeling for analysis and design of adsorption systems.

Amberlite XAD resin are widely used for modification with chelating materials due to its good physical and chemical properties such as porosity, high surface area, durability and purity. Many ligands, such as chronotropic acid [14], α-nitrozo β-naphtol [15], salicylic acid [16], pyrocathecol [17], 1-(2-pyridiazo)-2-naphtol [18], O-amino benzoic acid [19], 2-(methylthio) aniline [20], 3,4-dihydroxybenzoic acid [21], 2-aminothiophenol [22], and succinic acid [23] were covalently coupled with a polymer backbone through an azo (-N = N-) [24,25], methylene (-CH_2_-) [26] or other groups [27,28]. There are many reports of functionalized Amberlite XAD 2, 4 and 7 resins in this respect [29-37].

rhodium (Rh) is present at about 0.001 mg/L in the earth’s crust. Metallic rhodium metal is known for its stability in corrosive environments, physical beauty and unique chemical properties. It commands a premium price because of its low abundance in nature. Rhodium is now widely used in combination with platinum. Rhodium is commonly used for alloying platinum in thermocouples, crucibles, evaporating dishes, weighing boats windings for high-temperature furnaces. It finds applications as a coating material because of the hardness and luster of its surface. Because of its commercial importance, a wide variety of reagents have been proposed for preconcentration of Rh before spectrophotometric determination.

In this work, Amberlite XAD-4 alizarin red-s was prepared by chemically bonding to be used as an adsorbent. Alizarin red-s could form chelates with metallic ions on the surface of the resin. Adsorption of Rh(III) from aqueous solution and isotherm study using modified Amberlite XAD-4 was investigated under different experimental conditions to assess its affinity towards the chelator.

## Materials and methods

### Instruments

pH measurements were made with Metrohm model 744 (Switzerland) pH meter. IR spectra were recorded on a FT-IR spectrometer (Jasco/FT-IR-410) by KBr pellet method. Elemental analysis was carried out on an elemental analyzer from Thermo-Finnegan (Milan, Italy) model Flash EA. ICP-AES Varian, Vista-pro (Salt lake city, USA) was used for measuring the concentration of Rh (III). Thermo gravimetric analysis (TGA) was carried out by using TGA-50 H (Shimadzu, Japan).

### Reagents and solutions

Acetic acid, sodium acetate, sodium hydrogen phosphate, sodium dihydrogen phosphate, rhodium chloride, tin (II) chloride, hydrochloric acid, sulfuric acid, nitric acid, sodium nitrite, sodium hydroxide, alizarin red, and iodide-starch paper were products of Merck Co. (Darmstadt, Germany).

All of the solutions were prepared in demonized water using analytical grade reagents. The stock solution (500 mg/L) of Rh(III) was prepared by dissolving appropriate amounts of rhodium chloride respectively in demonized water. 10 mL 0.01 M acetic acid acetate buffer (pH = 3-5) and 0.01 M phosphate buffer (pH = 6-9) were used to adjust pH of the solutions, wherever suitable. Amberlite XAD-4 resin (surface area = 745 m^2^/g, pore diameter = 5 nm and bead size = 20-60 mesh) was obtained from Serve (Heidelberg, New York).

### Synthesis of chelating resin

Amberlite XAD-4 beads (5 g) were treated with 10 mL of concentrated HNO_3_ and 25 mL of concentrated H_2_SO_4_ and the mixture was stirred at 60°C for 1 hour on an oil bath. Then, the reaction mixture was poured into an ice water mixture. The nitrated resin was filtered, washed repeatedly with water until free from acid and then treated with a reducing mixture of 40 g of SnCl_2_, 45 mL of concentrated HCl and 50 mL of ethanol. The mixture was refluxed for 12 hours at 90°C. The solid precipitate was filtered and washed with water and 2 mol/L NaOH which released amino resin (R-NH_2_) from (RNH_3_)_2_ SnCl_6_ (R = resin matrix). The amino resin was first washed with 2 mol/L HCl and finally with distilled water to remove the excess HCl. It was suspended in an ice-water mixture (350 mL) and treated with 1 mol/L HCl and 1 mol/L NaNO_2_ (added in small aliquots of 1 mL) until the reaction mixture showed a permanent dark blue color with starch-iodide paper. The diazotized resin was filtered, washed with ice-cold water and reacted with alizarin red-s 0.03 mol in 30 mL 2 mol/L HCl, respectively. The reaction mixture was stirred at 0-3°C for 24 hours. Then, the resulting colored beads were filtered, washed with water and dried in air.

### Batch method

A sample solution (50 mL) containing (0.3 μg/mL) of Rh (III) was taken in a glass stopped bottle, after adjusting its pH to the optimum value. The 0.05 g of alizarin red S-Amberlite XAD-4 was added to the bottle and the mixture was shaken for optimum time. The resin was filtered and sobbed metal ion was eluted with 1.5 M HCl (10 mL). The concentration of metal ion in the elute solution was determined by ICP-AES. The wavelength of 343 nm was used for Rh determination.

### Isotherm studies of Rh (III) adsorption

Isotherm studies were carried out by adding a fixed amount of sorbent (0.05 g) to a series of beakers filled with 50 mL diluted solutions of Rh(III) (10-100 μg/mL) in 0.01 M acetate buffer and pH = 6.5. The beakers were sealed and placed in a water bath shaker and shaken at 200 rpm for 4 hours at 20°C. The beakers were then removed from the shaker, and the final concentration of Rh(III) in the solution was measured by flaming atomic absorption spectroscopy(FAAS). The amount of Rh(III) at equilibrium q_e_ (mg/g) on alizarin red S-Amberlite XAD-4 was calculated from the following equation:

(1) $$q_{e}{=}^{\text{V}}\text{/m}\left(C_{0}{-C}_{e}\right)$$

Where C_0_ and C_e_(mg/L) are initial and equilibrium concentrations of Rh(III), respectively; V (L) is the volume of the solution and m (g) is the mass of the adsorbent used.

### Adsorption parameters

#### Metal sorption as a function of pH

The degree of metal sorption at different pH values was determined by batch equilibration technique. A set of solutions (volume of each = 100 mL) containing 0.3 μg/mL of Rh(III) was taken. pH was adjusted in the range of 3-9 with 0.01 M acetate and/or phosphate buffer solutions. 0.1 g of alizarin red S-Amberlite XAD-4 was added to each solution and the mixture was shaken for 4 hours. The optimum pH for quantitative uptake of metal ions was ascertained by measuring Rh (III) content (by ICP-AES) in supernatant liquid and in the elute obtained by desorbing the metal ion from resin with 1.5 M hydrochloric acid (10 mL).

#### Sorption capacity

0.05 g of poly(AGE/IDA-co-DMAA)-grafted silica gel was stirred for 4 h. with 50 mL solution containing10-50 μg/mL of Rh(III) at optimum pH and 20°C. The metal ion concentration in the supernatant liquid was estimated by ICP-AES. The sorption capacity of the sorbent for the metal ion was ascertained from the difference between the metal ion concentration in the solution before and after the sorption.

#### Optimization of sorption time of rhodium ions

Alizarin red-s-amberliteXAD-4 (0.1 g) was shaken with 50 mL of solution containing 300 μg/mL of Rh (III) for different times (20, 60, 90, 120, 150 and 180 min) under optimum pH. After taking out the sorbent, concentration of rhodium ions in the solution was determined with ICP-AES using recommended batch method.

#### Adsorption isotherms

The Langmuir equation is given in the following form:

(2) $$q_{e}=q_{\text{max}}K_{L}C_{e}/\left(1+K_{L}C_{e}\right)$$

Where q_max_ is the maximum adsorption capacity corresponding to complete monolayer coverage on the surface (mg/g) and K_L_ is the Langmuir constant (L/mg). The equation (2) can be rearranged to a linear form:

(3) $$C_{e}/q_{e}=\left(1/q_{\text{max}}K_{L}\right)+\left(C_{e}/q_{\text{max}}\right)$$

**Figure 1:**
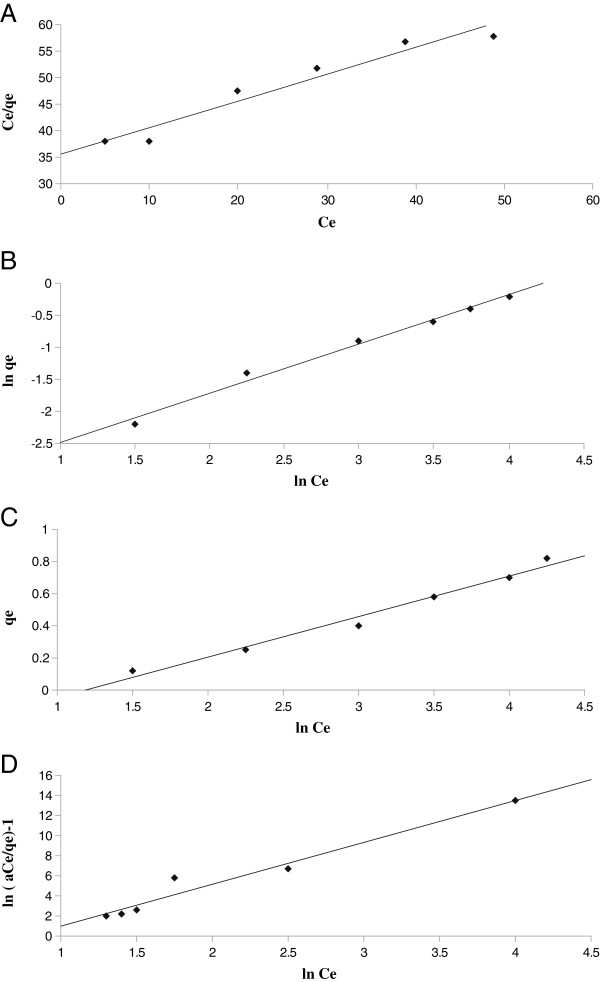
(A) Langmuir, (B) Freundlich, (C) Temkin and (D) Redlich-Peterson isotherms for Rh(III) adsorption onto Alizarin red S-Amberlite XAD-4.

The constants can be evaluated from the intercepts and the slopes of the linear plots of C_e_/q_e_ versus C_e_ (Figure 1A).

Conformation of the experimental data into Langmuir isotherm model indicates the homogeneous nature of Alizarin red S-Amberlite XAD-4 surface. Langmuir parameters calculated from Equation (3) are listed in Table 1.

**Table 1:** Isotherm parameters obtained by using non-linear method

				
	**Langmuir isotherm model**			
Temperature	q_max_(mg/g)	K_L_ (L/mg)	R_L_	R^2^
20°C	4.842	0.0147	0.5764	0.9986
	**Freundlich isotherm model**			
Temperature	K_F_ (mg/g)(L/mg)^1/n^	n	R^2^	
20°C	0.102	1.2816	0.9980	
	**Temkin isotherm model**			
Temperature	A (L/g)	B (J/mol)	b(J/mol)	R^2^
20°C	0.2668	0.7383	3299	0.9486
	**Redlich–Peterson isotherm model**			
g	B (dm^3^/mg)^g^	A (dm^3^/g)	R^2^	
0.2870	1.3569	0.2218	0.9980	

The essential characteristics of the Langmuir equation can be expressed in terms of a dimensionless separation factor, R_L_, defined as [38]:

(4) $$R_{L}=1/\left(1+K_{L}C_{0}\right)$$

Table 1 shows that the value of R_L_ (0.5764)is in the range of 0-1 at optimum pH which confirms the favorable uptake of the Rh(III).

The Freundlich equation is an empirical equation employed to the described heterogeneous systems, in which it is characterized by the heterogeneity factor 1/n. Hence, the empirical equation can be written as:

(5) $$q_{e}=K_{\text{F}}{C_{e}}^{1/\text{n}}$$

Where K_F_ is the Freundlich constant (mg/g) (L/mg) ^1/n^ and 1/n is the heterogeneity factor. A linear form of the Freundlich expression can be obtained by taking logarithms of the Equation (5):

(6) $${\text{lnq}}_{e}=\text{ln}\left(K_{\text{F}}\right)+1/\text{n ln}\left(C_{e}\right)$$

Therefore, a plot of ln(q_e_) versus ln(C_e_) (Figure 1B) enables the constant K_F_ and exponent 1/n to be determined. The Freundlich equation predicts that the Rh(III) concentration on the adsorbent will increase as long as there is an increase in Rh(III) concentration in the liquid.

The Temkin equation suggests a linear decrease of sorption energy as the degree of completion of the optional centers of an adsorbent is increased.

The Temkin isotherm has been generally applied in the following form:

(7) $$q_{e}=\frac{\text{RT}}{B}{\text{ln(AC}}_{e})$$

and can be linearized as:

(8) $$q_{e}=\text{B ln}\left(A\right)+\text{B ln}\left(C_{e}\right)$$

Where B = RT/b and b is the Temkin constant related to heat of sorption (J/mol). A is the Temkin isotherm constant (L/g), R is the gas constant (8.314 J/mol K) and T is the absolute temperature (K). Therefore plotting q_e_ versus ln(C_e_) (Figure 1C) enables one to determine the constants A and B. Temkin parameters calculated from Equations (7 and 8) are listed in Table 1.

The Redlich–Peterson isotherm contains three parameters and incorporates the features of the Langmuir and the Freundlich isotherms. The Redlich–Peterson isotherm has a linear dependence on concentration in the numerator and an exponential function in the denominator. It can be described as follows:

(9) $$q_{e}=\frac{{\text{AC}}_{e}}{1+{\text{BC}}_{e}^{g}}$$

It has three isotherm constants, namely, A, B, and *g* (0 < *g* < 1), which characterize the isotherm. The limiting behavior can be summarized as follows:

Where *g* =1:

(10) $$q_{e}=\frac{{\text{AC}}_{e}}{1+{\text{BC}}_{e}}$$

i.e. the Langmuir form results.

Where constants *A* and *B* are much greater than unity [39]:

(11) $$q_{e}=(\frac{A}{B}){C_{e}}^{1-g}$$

i.e. the Freundlich form results.

Where *g* = 0:

(12) $$q_{e}=(\frac{A}{1+B})C_{e}$$

i.e. the Henry’s Law form results.

Eq. (9) can be converted to a linear form by taking logarithms:

(13) $$\text{ln}\left(A\frac{C_{e}}{q_{e}}-1\right)=\text{gln}\left(C_{e}\right)+\text{ln}\left(B\right)$$

Three isotherm constants, A, B, and *g* can be evaluated from the linear plot represented by Eq. (13) using a trial and error procedure. It was developed to determine the isotherm parameters by optimization routine to maximize the coefficient of determination, R^2^, for a series of values of A for the linear regression of ln(C_e_) on ln[*A*(C_e_/q_e_) − 1] and to obtain the best value of A which yields a maximum ‘optimized’ value of *R*^*2*^ using the solver add-in with Microsoft’s spreadsheet, Microsoft Excel(Figure 1D).

Scat chard analysis was employed to further analyze the binding isotherms, which is an approximate model commonly used in SPE (Solid Phase Extraction) characterization. The Scat chard equation can be expressed as, Q/C = (Q_max_–Q)/K_d_, where C (μmol/mL) is the equilibrium concentration of rhodium; Q (μmol/g) is the equilibrium adsorption amount at each concentration Q_max_ (μmol/g) is the maximum adsorption amount; and K_d_ (μmol/mL) is the equilibrium dissociation constant at binding sites.

## Results

### IR spectrum

**Figure 2:**
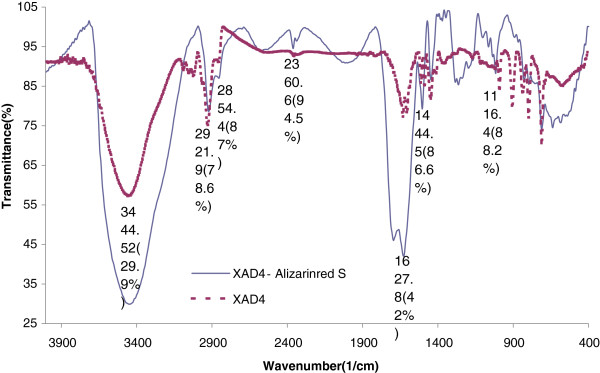
FT-IR spectrum of Alizarin red S-Amberlite XAD-4.

In Figure 2 the experimental FTIR spectrum of alizarin red-s loaded Amberlite XAD-4 is compared with that of free Amberlite XAD-4. There are two additional bands at 1638/cm and 3432/cm which appear to originate due to modification of N = N and O-H, respectively (Figure 2).

### Thermal analysis

TGA of the resins shows two step weight losses up to 510°C. The weight loss up to 130°C was due to the water molecules in the polymer. The major weight loss after 290°C is due to the dissociation of chemically immobilized moiety and the polymeric matrix.

**Figure 3:**
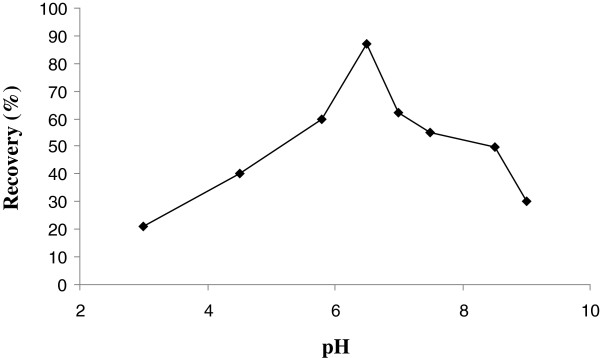
Effect of pH sorption of Rh(III) onto Alizarin red S-Amberlite XAD-4.

The optimum pH range for the sorption of Rh (III) is shown in Figure 3. The maximum recovery was 88.3% at pH = 6.5.

**Figure 4:**
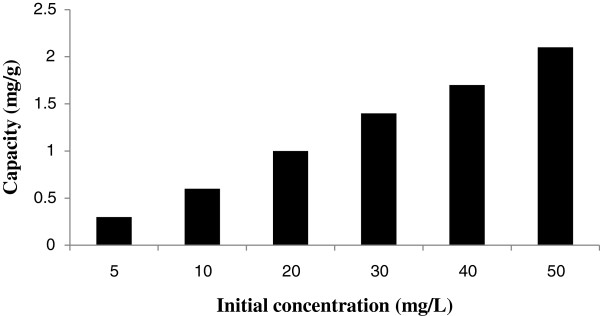
Effect of initial concentration of the Rh(III) in the solution on sorption capacity.

The saturated adsorption capacity of the resin is shown in Figure 4. This figure indicates the effect of initial concentration of the Rh(III) in the solution it’s on sorption by DMAA-AGE/IDA- grafted silica gel. The capacity goes up with increasing initial concentration of the Rh(III) in the solution (2.1 mg/g at initial concentration of 50 mg/L).

### Stability and reusability of the adsorbent resin

Rh(III) was absorbed and desorbed on 1 g of the resin several times. It was found that sorption capacity of resin after 10 cycles of its equilibration with Rh(III), changes less than 5%. Therefore, repeated use of the resin is feasible. The resin cartridge after loading it with samples can be readily regenerated with 1.5 M HCl. The sorption capacity of the resin stored for more than 6 months under ambient conditions has been found to be practically unchanged.

**Figure 5:**
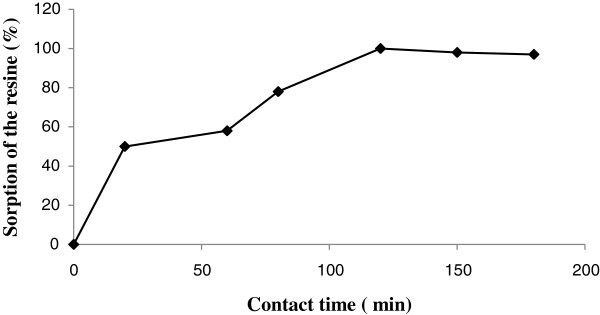
Sorption as a function of contact time.

The sorption as a function of contact time for all metal ions is shown in Figure 5. Less than 20 min shaking was required for about 50% sorption. The profile of rhodium uptake on this sorbent reflects good accessibility of the chelating sites in the Alizarin red-s-Amberlite XAD-4.

### Optimization of desorption time of rhodium ions

**Figure 6:**
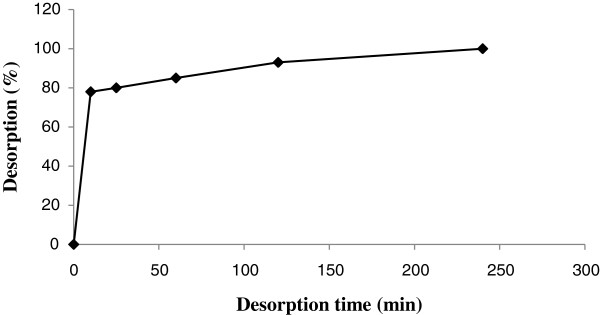
Desorption as a function of contact time.

For stripping off the bounded Rh(III) on modified Amberlite XAD-4, 1.5 M HCl was applied in different times (10-240 min) (see Figure 6). Less than 10 minutes shaking was required for about 78% desorption.

The Redlich–Peterson isotherm constants, A, B, and g as well as the coefficient of determination, R^2^, for the sorption of Rh(III) onto Alizarin red S-Amberlite XAD-4 using the linear regression is shown in Table 1. It can be seen that the values of (g) were close to unity, which means that the isotherms are approaching the Langmuir form and not the Freundlich isotherm. The result shows that the Langmuir isotherm best-fit the equilibrium data for adsorption of Rh(III) on Alizarin red S-Amberlite XAD-4.

### Scat chard analysis

**Figure 7:**
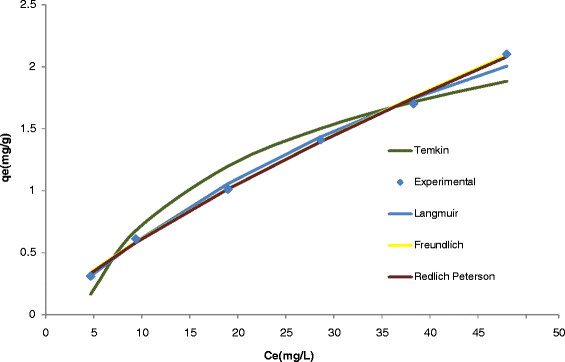
Scatchard plots of Rh(III) adsorption onto Alizarin red S-Amberlite XAD-4.

Figure 7 shows the Scat chard plots of binding of rhodium to the sorbent. It is clear that the Scat chard plot for sorbent is a single straight line. The linear regression equation was Q/C = –1.3169Q + 27.222 (R^2^ = 0.9239), suggesting that the homogeneous recognition sites for rhodium were formed in the SPE(Solid Phase Extraction)sorbent. From the slope (–1.3169 (1/K_d_)) and intercept (27.222 (Q_max_/K_d_)), K_d_ and Q_max_ for the affinity binding sites were calculated to be 0.76 μmol/mL and 20.67 μmol/g, respectively.

### Application of method

Alizarin red S -Amberlite XAD-4 was used to preconcentrate and measurement ofRh(III) ions in tap water (Tehran) and spring water (Bagh e Feiz,Tehran). The pH of water sample was adjusted to the optimum pH = 6.5. Solid phase extraction with Alizarin red-s -Amberlite XAD-4 coupled with ICP-AES was applied to determinRh(III) concentration in water sample. Since no Rh(III) was detected in the water samples, 100 mL water sample was spiked with 0.02 and 0.08 mg of Rh(III) before subjecting it to the recommended procedure. The results are shown in Table 2.

**Table 2:** Results obtained for Rh(III)measurement in tap water (I) and spring water (II)

**Found (without spiking of Rh(III))**	**I**	**II**
	**N.D.**	**N.D.**
Added Rh(III) (μg/mL)	0.2	0.8
Found Rh(III), after preconcentration (μg/mL)	1.6	6.7
Preconcentration factor	10	10
Recovery (%)	80.0	83.7
Standard deviation	0.11	0.15
Relative standard deviation (%) ^a^	6.87	2.24

a: For three determinations.

## Discussion

The experimental FT-IR spectrum and thermal analysis (TGA) show this resin was satisfactory for adsorption of rhodium ion. In pH = 6.5, the results indicated 88% recovery and at 15 min (Figure 5) the adsorption of resin was about 50%. The equilibrium data and parameters of Rh(III) adsorption on modified resin were analyzed by Langmuir, Freundlich, Temkin and Redlich–Peterson models. The experimental adsorption isotherm was in good concordance with Langmuir and Freundlich models (R^2^ > 0.998) and based on the Langmuir isotherm the maximum amount of adsorption (q_max_) was 4.842 mg/g.

**Figure 8:**
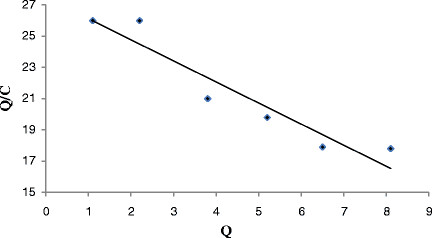
Isotherms obtained using the non-linear method for the adsorption of Rh (III) onto Alizarin red S -Amberlite XAD-4 at a temperature of 293 K.

A new resin was synthesized by coupling of Amberlite XAD-4 with Alizarin red S. The synthesis of the resin is simple and economical. The resin had a good potential for enrichment of trace amount of Rh(III) from large sample volumes. The Rh(III) adsorption was due to immobilized ligand-metal ion interactions. The resins also presented the advantage of high adsorption capacity, good reusability and high chemical stability. The sorption/desorption of metal ion took place in moderate time, making the analytical procedure reasonably fast. Finally, the different isotherms were tested for their ability to correlate with the experimental results by comparing theoretical plots of each isotherm with the experimental data for the adsorption of rhodium ions on Alizarin red S -Amberlite XAD-4 at 293 K in Figure 8. In this graph, the amount of rhodium sobbed per unit mass of Alizarin red S -Amberlite XAD-4, q_e_, is plotted against the concentration of rhodium remaining in solution, C_e_ and the good fit of the Redlich–Peterson and Langmuir isotherms were not the same even when the coefficient of determinations was high for both isotherms.

The results in Table 1 indicated that this work is better than other isotherms modeling [40].

The results about application of this work in real sample and environmental studies, also demonstrated the applicability of the procedure for rhodium determination in real samples with high recovery (>80%).

## Competing interests

The authors declare that they have no competing interests.

## Authors’ contributions

HSK conceived of the study and participated in coordination. HAP participated in the statistical analysis of data. HH and MT participated in graphical and tables preparation. MTM participated the experimental studies. All authors read and approved the final manuscript.
